# Quantifying Antarctic krill connectivity across the West Antarctic Peninsula and its role in large-scale *Pygoscelis* penguin population dynamics

**DOI:** 10.1038/s41598-023-39105-6

**Published:** 2023-07-26

**Authors:** Katherine L. Gallagher, Michael S. Dinniman, Heather J. Lynch

**Affiliations:** 1 Institute for Advanced Computational Sciences, Stony Brook University, Stony Brook, NY 11794 USA; 2 Department of Ocean and Earth Sciences, Old Dominion University, Norfolk, VA 23529 USA; 3 Department of Ecology & Evolution, Stony Brook University, Stony Brook, NY 11790 USA

**Keywords:** Biogeography, Population dynamics, Physical oceanography

## Abstract

Antarctic krill (*Euphausia superba*) are considered a keystone species for higher trophic level predators along the West Antarctic Peninsula (WAP) during the austral summer. The connectivity of krill may play a critical role in predator biogeography, especially for central-place foragers such as the *Pygoscelis* spp. penguins that breed along the WAP during the austral summer. Antarctic krill are also heavily fished commercially; therefore, understanding population connectivity of krill is critical to effective management. Here, we used a physical ocean model to examine adult krill connectivity in this region using simulated krill with realistic diel vertical migration behaviors across four austral summers. Our results indicate that krill north and south of Low Island and the southern Bransfield Strait are nearly isolated from each other and that persistent current features play a role in this lack of inter-region connectivity. Transit and entrainment times were not correlated with penguin populations at the large spatial scales examined. However, long transit times and reduced entrainment correlate spatially with the areas where krill fishing is most intense, which heightens the risk that krill fishing may lead to limited krill availability for predators.

## Introduction

Antarctic krill (*Euphausia superba;* henceforth referred to as krill) abundance along the Western Antarctic Peninsula (WAP) is highly dynamic on both spatial and temporal scales^1–6^, with cycles peaking approximately every 4–6 years^7,8^. Recruitment of juvenile krill in the region has been linked to the extent of sea ice in the previous winter^9–12^. Ice dynamics in the spring also play a role in phytoplankton distributions, size, and availability to krill recruits^13,14^. These dynamics have been linked to shifts in large scale climate oscillations like the Southern Annular Mode and El Nino Southern Oscillation^6,13,15^. Furthermore, a growing krill fishery has added additional stressors to a delicate system on fine spatial scales^16–21^.

**Figure 1:**
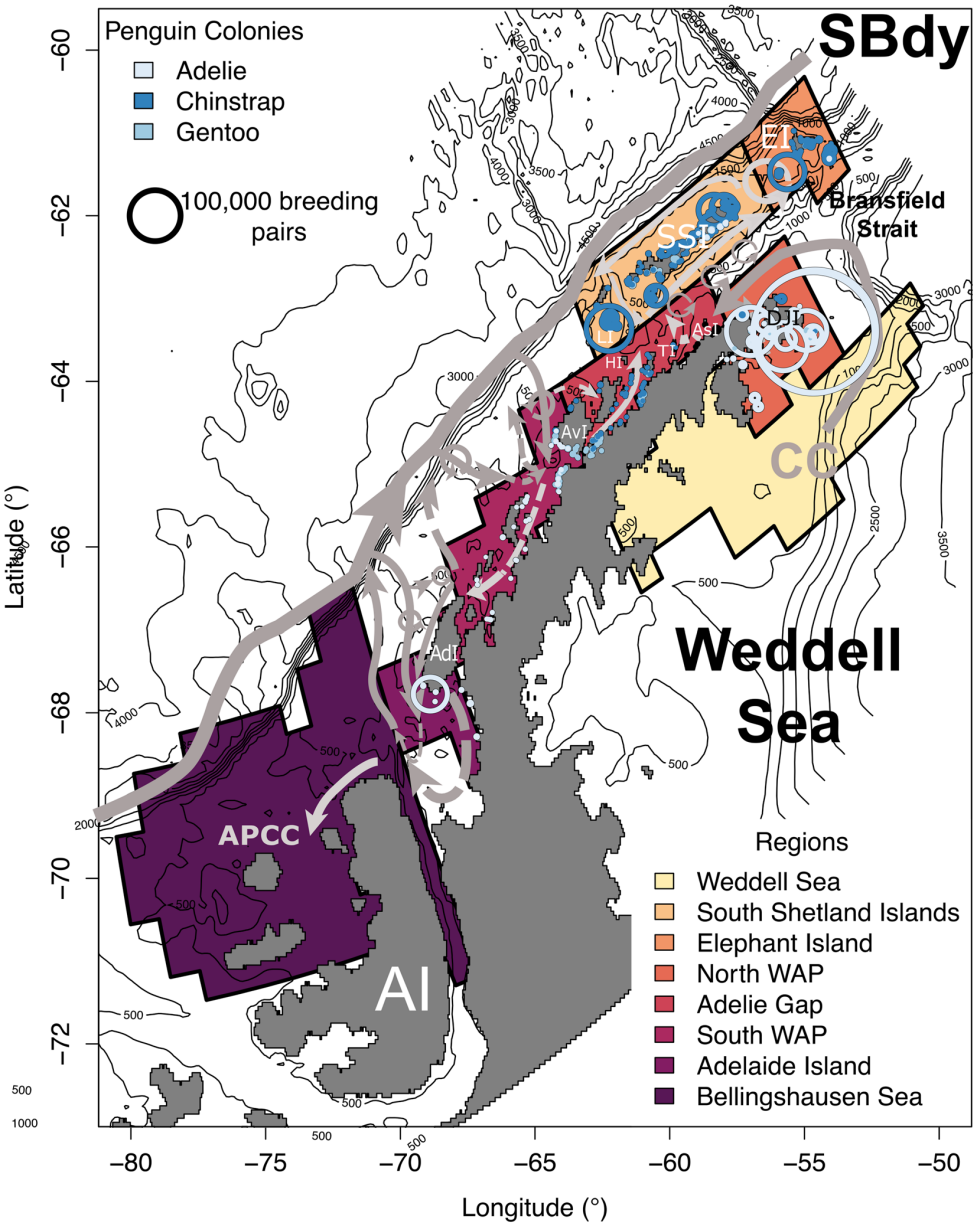
Map of the Antarctic Peninsula, including land masks and bathymetry from the Regional Ocean Modeling System (ROMS). Boxes illustrate the 8 regions used to estimate connectivity: Bellingshausen Sea, Adelaide Island, South Western Antarctic Peninsula (WAP), Adélie Gap, North WAP, South Shetland Islands, Elephant Island, and Weddell Sea. Grey arrows illustrate WAP circulation from Moffat & Meredith (2018)^32^. Abbreviations indicate relevant current systems and islands: *SBdy* Southern ACC Boundary, *APCC* Antarctic Peninsula Coastal Current, *CC* Antarctic Coastal Current, *EI* Elephant Island, *SSI* South Shetland Islands, *DJI* D’Urville and Joinville Islands, *AsI* Astrolabe Island, *TI* Tower Island, *HI* Hoseason Island, *LI* Low Island, *AvI* Anvers Island, *AdI* Adelaide Island, *AI* Alexander Island. Circles illustrate *Pygoscelis* penguin colonies with circle size indicating colony size.

Observations and modeling experiments suggest that krill spawning off the continental shelf in the Bellingshausen Sea supplies juvenile and adult krill to the central and southern regions of the Peninsula, as well as to the South Shetland Islands and islands to the north of the WAP^22–26^. Spawning in the Bellingshausen Sea does not, however, appear to serve as a source of krill to the northern tip of the WAP. Ocean currents around the tip of the WAP may isolate this region from the rest of the Peninsula, which relies instead on krill spawning in the Weddell Sea through the Coastal Current (CC; Fig. 1)^27–29^. Krill eggs have also been observed along portions on or near the continental shelf break in the northern WAP, where the CC and other currents within the Bransfield Strait would keep larval krill in this region, or advect them to points downstream such as South Georgia via the Antarctic Circumpolar Current (ACC)^27,30^.

While larval krill are generally found on or near the continental shelf break^30^, juvenile and non-spawning adult krill are found in the coastal waters of the continental shelf during the austral summer^25,30^. Post-larval krill distributions along the WAP during this critical season are well studied^2,7,25,31^, however, the connectivity within the krill population during the austral summer is poorly understood. Because krill distributions in this region are spatially heterogeneous^32^, connectivity among regions can have an important influence on krill availability not only for predators, such as breeding *Pygoscelis* penguins that feed primarily on krill^33^, but also for a growing krill fishery^17,19,34,35^.

### Penguins along the WAP

All three *Pygoscelis* species, Adélie (*Pygoscelis adeliae*), gentoo (*P. papua*), and chinstrap (*P. antarcticus*) breed along the WAP^36–39^. Of the three species, Adélie penguins have the strongest ties to sea ice, which they utilize as critical overwintering habitat^40^. Adélies are also the first species to return to initiate breeding, and they often contend with snow and/or ice-covered habitat and melt during the early breeding season^40–42^. Wetter conditions that make it challenging to keep eggs dry during incubation and cause heat loss among downy chicks has been negatively correlated with Adélie penguin breeding success^43^. Gentoo and chinstrap penguins, however, initiate breeding later than Adélies, potentially avoiding the most challenging snow conditions^42^. Moreover, gentoos and chinstraps, whose core breeding habitat is further north in the sub-Antarctic, are better adapted to milder conditions^37,44^. During the critical breeding period, all three species act as central place foragers and have similar diets^33,42^. While gentoos have higher diet plasticity, all three species primarily consume krill during the chick-rearing period^33^. Due to differences in clutch initiation, breeding pygoscelid penguins consume differing amounts of krill in different periods of the austral summer, with Adélies consuming more krill earlier in the season than their counterparts^42^.

In addition to these ecological differences, the biogeography and population status of all three species vary widely across the WAP^45,46^. The largest Adélie penguin colonies are found in the northernmost portions of the Peninsula where their populations are also relatively stable in contrast to colonies elsewhere in the region^45,47^. Chinstrap colonies are more common in the South Shetland Islands, where colony size is shrinking, and are present only in extremely small numbers south of Anvers Island^45^. Gentoo penguins, however, have actively expanded their range south over the past three decades, especially in the region around Anvers Island^37^. Gentoo colonies tend to be smaller but colonies are numerous in and around the Gerlache Strait and south of Anvers Island (Fig. 1). Small gentoo colonies are also found throughout the South Shetland and Elephant Islands, as well as near the northern tip of the WAP (Fig. 1).

### Antarctic krill fishing

The Antarctic krill fishery has been putting increasing pressure on local krill predator populations, especially along the WAP where fishing is concentrated in the Bransfield Strait (Fig. 1) between the South Shetland Islands and the Peninsula^17–19^. The fishery is currently managed by the Commission for the Conservation of Antarctic Marine Living Resources (CCAMLR), which takes an ecosystem management approach due to the krill’s critical role in Southern Ocean ecosystems. Despite efforts such as catch limits and improved reporting metrics, recent work suggests that penguin reproductive success decreases with increasing fishing activity^17^. Furthermore, krill abundance along the WAP has been linked to pregnancy rates in humpback whales, suggesting prey limitation^20^. The ramifications of intense fishing pressure near predator hotspots remain poorly understood. As a result, several recent studies have highlighted the need for management on small spatial scales^18,19,21^.

Outside CCAMLR, the Association of Responsible Krill (ARK) harvesting companies implemented voluntary fishing restrictions near penguin colonies in 2018^19,48^. These restrictions are, for the most part, only in place during the austral summer within 40 km of penguin breeding colonies^48,49^. While these voluntary restrictions may improve prey accessibility to predators locally, they will not improve krill availability on larger spatial scales if penguin colonies are relying on upstream sources of krill that are heavily fished.

### Krill connectivity and penguin dynamics

The connectivity of krill in both the context of prey availability to penguins during the austral summer and fisheries management has recently been examined by Trathan et al. using three-day averages of 3D velocities from a high-resolution oceanographic model^19^. Using passive particles, they highlighted the importance of the CC and currents within the Bransfield Current System (BCS) in providing krill to nearby chinstrap penguin colonies along the South Shetland Islands^19,35^. The assumption that krill are passive drifters in the horizontal, however, ignores a critical krill behavior—diel vertical migration (DVM)—that krill perform to avoid visual predators during the day and consume phytoplankton in productive surface waters at night^50^. Previous modeling studies used Lagrangian particles with DVM to study its impact on transport pathways of larval krill spawned off the continental shelf in the Bellingshausen Sea and illustrated that DVM does not significantly alter pathways of these larval krill^22^. However, recent work has highlighted the impact of DVM on retention of krill near predator foraging grounds showing the importance of DVM in certain locations with strong vertical gradients in circulation^51^.

Here, we build upon these previous findings and examine krill population connectivity along the WAP continental shelf during the austral summer across the entire region to determine the origins and transport pathways of krill advected near all pygoscelid penguin colonies in the region. Using a 1.5 km horizontal-resolution physical ocean model that includes tidal forcing, we simulate krill movement, including DVM, through the austral summer across the entire Antarctic Peninsula region from the Marguerite Trough to the Weddell Sea. Based on known current features along the WAP^27^, we hypothesized that krill advected close to penguin colonies south of the Bransfield Strait will originate from the Bellingshausen Sea, while krill advected near penguin colonies north of this region will originate from the Weddell Sea, with little interaction between these two groups. In addition, we hypothesized that the addition of DVM to simulated krill will make connectivity between regions along the coastal WAP more consistent since the addition of DVM will allow krill to interact with the generally slower, more consistent currents present in deeper waters. We also examined the timing of regional connectivity through two metrics: how long simulated krill spend in each of our study regions and how long it takes them to travel to these regions from elsewhere. We hypothesized that regions where krill spent more time and had shorter transit times (meaning that they spent less time traveling to these regions) would have larger penguin populations.

Because *Pygoscelis* population dynamics vary so widely across the WAP, we hypothesized that the source and pathways of krill to different regions would explain penguin population trends within each area of interest. Evidence supporting our hypotheses would suggest that krill connectivity, and the timing of that connectivity, may explain the dichotomy between areas north and south of the Bransfield Strait and, furthermore, may influence the efficacy of regional krill fishing closures.

## Methods

### Regional ocean modeling system

To test how krill along the WAP are connected, we used an updated regional WAP implementation of the Regional Ocean Modeling System (ROMS)^52–54^. Simulation descriptions are provided in Supplementary Text A. This version of the model has a 1.5 km horizontal resolution and 24 vertical terrain-following layers. Dynamic sea ice and the interactions between floating ice shelves and the underlying waters are included^55,56^. We simulated four austral summers from November to March: 2008–2009, 2009–2010, 2018–2019, and 2019–2020. We refer to each of these summers as a season, using the year in which the summer started to differentiate the simulations. Tidal forcing is from the CATS2008 regional Antarctic tidal model, with nodal corrections applied as necessary^57^. Atmospheric forcing is from the Antarctic Mesoscale Prediction System (AMPS)^58^. Spatial resolution of AMPS varied between 15 and 20 km for the 2008 and 2009 seasons and increased to 8 km for the 2018 and 2019 seasons.

### Modeling krill behavior

Simulated particles served as a proxy for krill (henceforth referred to as ‘simulated krill’) and were seeded on an approximately 8 km grid throughout the study region (Fig. S1). A total of 16 weekly release events occurred, starting on 1 November and ending in mid-February of each simulation. Simulated krill were released every 7 days and were tracked for at least 30 days. To mimic the effect of vertical turbulence (which is parameterized in ROMS), simulated krill positions included a vertical random walk^59,60^. Simulated krill were advected at every model baroclinic time step (50 s) and positions were saved hourly.

To simulate krill behavior, DVM was added to passive particles within ROMS. This behavior was added to ROMS particles previously to simulate both zooplankton^61^ and krill^51^. DVM in ROMS was based on local solar angle. When the sun was above the horizon, downward velocities were added to the advective and random vertical velocities in the model if the simulated krill was above a target depth. Inversely, when the sun was below the horizon, an upward velocity was added if the simulated krill was below a target depth (10 m).

Simulated krill migration depths were based on previously published observations of krill DVM along the WAP (Table S1) and *Pygoscelis* penguin foraging depths (Table S2). Based on these observations, simulated krill DVM occurred between a minimum depth of 10 m and a maximum depth that varied between simulations (25, 50, 75, 100, or 150 m). Vertical migration speed was determined using observed krill swimming speeds in body lengths (BL) per second^62^. The mean BL of krill in swarms in the northern WAP and observed in *Pygoscelis* penguin diets near Anvers Island is approximately 43 mm^63,64^. With a mean vertical swimming speed of 0.335 BL s^−1^ in late spring^62^, vertical migration speed of simulated krill was set to 0.014 m s^−1^. Reverse DVM (krill spending time near the surface during the day and migrating down at night) was not considered here since it is not a common krill behavior in the coastal ocean^61,65,66^. We also calculated the difference in the number of particles, as well as percent difference, in our connectivity metrics (see "Regional connectivity") between simulated krill with and without DVM. These non-migrating simulated krill were subject to the same vertical random walk and were released at 25, 50, 75, 100, and 150 m.

In implementing this krill movement model within ROMS, we made three key assumptions. The first assumption was that krill only swam vertically. This assumption has been made in previous krill movement models^67–71^. In addition, krill movement directionality is largely unknown, and a Brownian-like movement model would yield no net horizontal movement on the horizontal scale of ROMS. While simulated directed swimming has been shown to influence krill transport using randomly sampled directions, this work was done without the addition of DVM. Therefore, to determine the impacts of the addition of DVM and compare to previous work that considered krill to be passive drifters, we selected to only model DVM. Our second assumption was that krill were regularly performing DVM to the depths selected. Krill DVM along the WAP is highly variable (Table S1) and has been shown to stop during certain points of the year, such as on the summer solstice^65,72^. We treat all krill released within a single season with DVM behaviors as a single population to account for a portion of the variability in DVM. Non-migrating simulated krill released at different depths were treated as a separate population to test the impacts of DVM on connectivity pathways. The third assumption made in these simulations was that krill were homogenously distributed in the environment upon release. Krill are heterogeneously distributed throughout the WAP^1,2,5–7,73,74^. The true distribution and biomass of krill, and drivers in changes to these quantities, in the WAP is still an active area of research with many remaining uncertainties^73,75^. Therefore, since the real distribution of krill is unknown across the broad spatial scales considered here, we chose to assume that krill were homogenously distributed.

### Regional connectivity

Connectivity was examined between the following regions: Adelaide Island, South WAP, the Adélie Gap, North WAP, the South Shetland Islands, and Elephant Island (Fig. 1). These regions were based on the location of *Pygoscelis* penguin colonies from the Mapping Application for Penguin Populations and Projected Dynamics (MAPPPD)^76^. All regions were named based on geographic location, except for the Adélie Gap. This region has been defined previously between Astrolabe and Anvers Islands. Here, only a handful of small chinstrap and gentoo colonies are present and no Adélie penguin colonies are found (Fig. 1)^77–80^.

Only areas approximately 40 km from these colonies were considered, as this approximates the maximum penguin summer foraging range for most colonies on the peninsula due to their size^81^. These areas were selected using a 1600 km^2^ grid generated across the model domain and selecting grid cells with extant penguin colonies within and adjacent to them. Two additional regions were considered as potential sources for krill: the north Weddell Sea and the coastal Bellingshausen Sea (Fig. 1). In these potential source regions, all areas where simulated krill were released are considered, regardless of distance to shore. We also considered simulated krill released from outside of these regions as external, non-coastal sources of krill. We refer to these krill as originating from offshore waters. Any simulated krill released in model water points under ice shelves were excluded.

To determine which simulated krill interacted with which regions, we noted when simulated krill were within each region using *point.in.polygon* in the R package ‘sp’^82^. From these trajectories, we noted the origin of the simulated krill, transit time to each region, and how long it was present in the region of interest. Transit time was only examined for simulated krill not originating within the region of interest. Similarly, how long simulated krill were present within each region was considered for simulated krill released within the region and elsewhere separately. We examined the distribution of these metrics for all release events that occurred within the chick-rearing period (December–March) across DVM behaviors and seasons. We also examined differences in connectivity metrics within simulated krill DVM behaviors and seasons. Metrics were compared using pairwise Wilcox tests with Bonferroni correction.

Daily average currents from ROMS and simulated krill paths were examined as possible mechanisms of connectivity. Currents were interpolated to 10, 25, 50, 75, 100, and 150 m to match simulated DVM behaviors and averaged within the chick-rearing period across all seasons at each depth. To visualize simulated krill trajectories throughout the simulations, 20% of released particles with one of five DVM behaviors were randomly selected within each season to account for variability in these behaviors. Tracks were visualized across all seasons.

### *Pygoscelis* penguin colony size

For each region, the number of penguin nests for each *Pygoscelis* penguin colony was summed to determine the total number of birds present within each region using the 2019 projections from MAPPPD. We used MAPPPD projected populations for each colony within the regions and correlated population sizes to our connectivity metrics within each region. Spearman’s rank correlations were used to compare median connectivity timing metrics to penguin populations, both across species and at the species level, using the function *cor.test* in the R *stats* package^83^. Confidence intervals were generated using the z-transformation method within the *SpearmanRho* function in the *DescTools* package^84^.

## Results

To examine the connectivity of krill between our study regions, we examined three metrics: (1) the number of simulated krill that entered each region that originated elsewhere; (2) the transit time of simulated krill to different regions; and (3) the time simulated krill spent in each region. Below, we review these calculated metrics ("Sources of simulated krill" and "Timing of Connectivity") and discuss the physical oceanographic phenomena responsible for these patterns ("Features driving connectivity"). We then correlate these metrics and phenomena to penguin colony size ("Penguins impacted by persistent features").

### Sources of simulated krill

**Figure 2:**
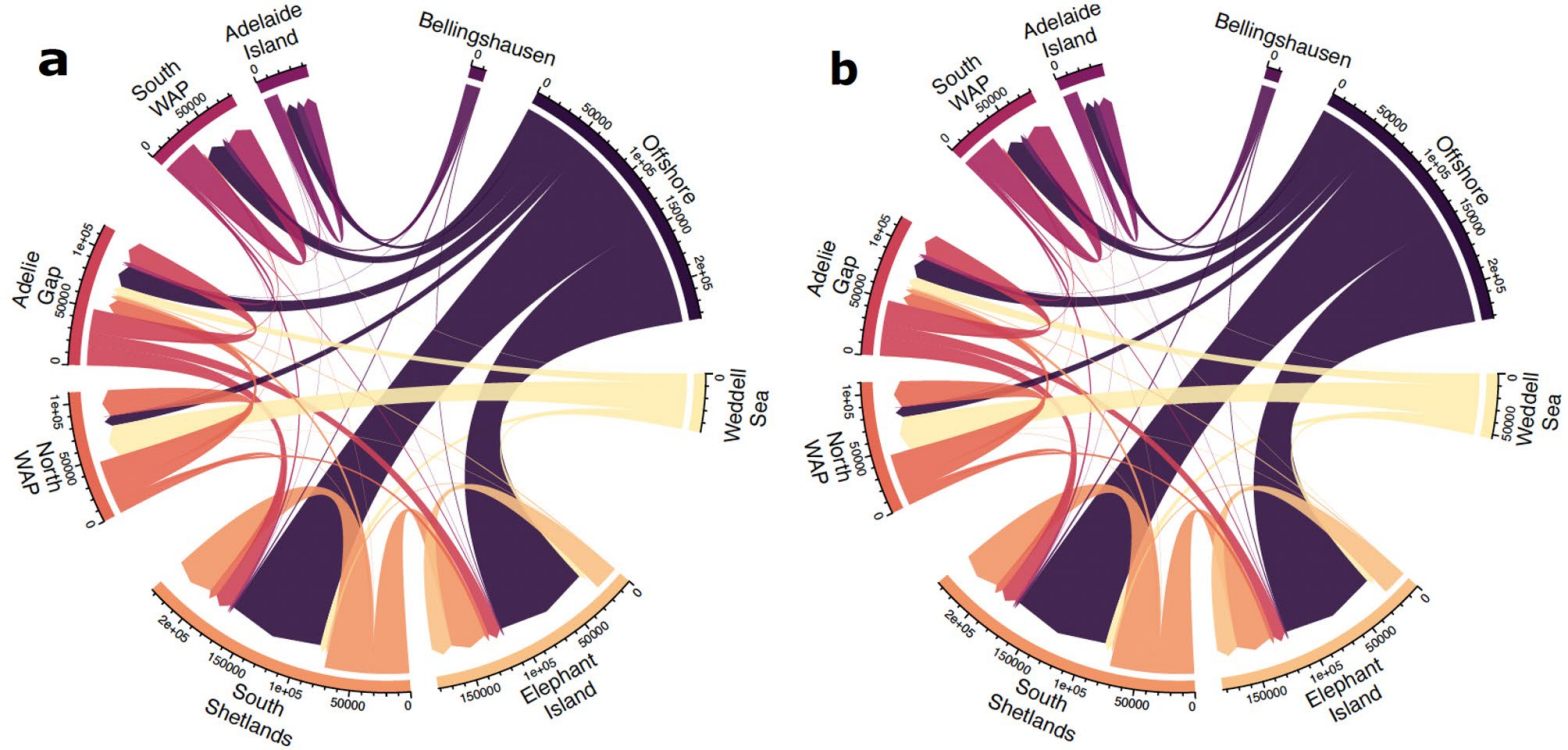
Chord diagrams illustrating interannual averages of the number of krill that entered each of the study regions from source regions (Bellingshausen, Offshore, and the Weddell Sea) or other study regions (all other regions) for simulated krill with (**a**) and without (**b**) diel vertical migration.

The transport pathways between simulated krill with and without DVM were very similar, with the interannual averages in the number of krill that entered the study regions varying by at most 12% across origins (Fig. 2, Fig. S2). The greatest differences were more than 5% increases in the number of simulated krill entering the South WAP from points north (Weddell Sea, South Shetlands, and Elephant Islands) and the North WAP from the South WAP when simulated krill did not perform DVM (Fig. S2). Across DVM behaviors and years, we found that the presence of DVM allowed krill released in some regions to travel farther. For example, more krill performing DVM released in the Weddell Sea enters waters around the Adélie Gap and to points south (South WAP and Adelaide Island) in comparison to when DVM was absent (Fig. S3). The presence of DVM also increased the number of krill from the Adélie Gap advected to the South Shetland and Elephant Islands (Fig. S3). The absence of DVM also resulted in more simulated krill from the Weddell Sea moving into the South Shetland and Elephant Islands (Fig. S3). However, these differences were less than 1000 simulated krill across all behaviors and years, which represents a small fraction of the simulated krill that ultimately interacted with these regions (Fig. 2, Figs. S3–8). Therefore, the presence of DVM behaviors did not significantly alter transport pathways between our study regions. Below, we discuss the major pathways of simulated krill performing DVM that entered each region.

Of our study regions, the fewest simulated krill entered the waters around Adelaide Island. Most were released there, with the Bellingshausen Sea and offshore waters serving as primary external sources (Fig. 2). A small fraction of simulated krill from the South WAP also transited to Adelaide Island (Fig. 2). Slightly more simulated krill moved into the South WAP than Adelaide Island, with most being released within the region or originating from offshore waters (Fig. 2). Few simulated krill that entered the South WAP region originated from other study regions (Fig. 2).

Simulated krill that interacted with the Adélie Gap originated from all study regions. The contribution of external regions, listed from largest to smallest source, were offshore waters, North WAP, Weddell Sea, South Shetland Islands, South WAP, Elephant Island, and Bellingshausen Sea (Fig. 2). In contrast, simulated krill that entered the North WAP originated primarily from the Weddell Sea, followed by offshore regions (Fig. 2). Very few simulated krill that entered this region originated from the Adélie Gap, the South Shetland Islands, or Elephant Island (Fig. 2).

The island regions around the South Shetland and Elephant Islands both received krill from all other regions (Fig. 2). Both received the most simulated krill from offshore waters. The Adélie Gap served as the second greatest course of simulated krill to the South Shetland Islands, followed by the North WAP. The Bellingshausen Sea, South WAP, and Elephant Island regions all served as small sources of simulated krill to this region (Fig. 2). The South Shetland Islands served as the second highest source of simulated krill to the Elephant Islands after the offshore regions. The Adélie Gap also served as a source of simulated krill to the Elephant Islands, while regions south of the Adélie Gap only supplied trivial amounts of krill to the region (Fig. 2).

### Timing of connectivity

**Figure 3:**
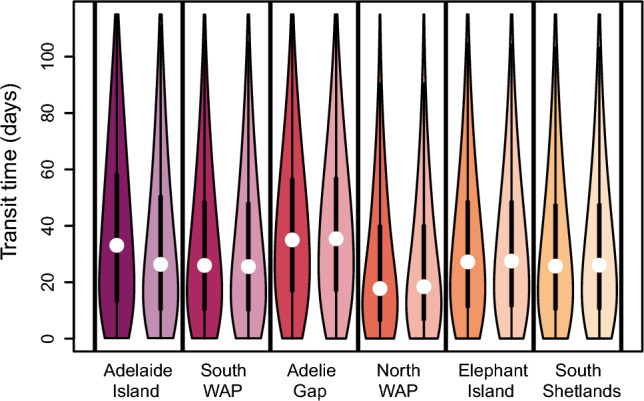
Violin plots illustrating the distribution of transit times in days simulated krill took to travel to our study regions across diel vertical migration behaviors and model years. White points indicate the median transit time and heavy black lines illustrate the interquartile range. Width of the bars indicate the distribution of the data, with wider bars indicating more data is present at that value. Light colors illustrate the transit times of simulated krill that did not perform DVM and dark colors indicate the transit times of simulated krill that did perform DVM.

We calculated two metrics measuring the timing of connectivity: how long simulated krill took to enter a region for the first time, and how long krill spent in each region. For simulated krill not released within a region, we examined the time taken for the simulated krill to be transported to that region (Fig. 3). Simulated krill with and without DVM behavior had nearly identical median transit times, except for Adelaide Island where simulated krill without DVM entered the region 5 days sooner than simulated krill performing DVM (Fig. 3). DVM depth and year also did not significantly impact transit times (Fig. S9).

Simulated krill had the longest median transit times to the Adélie Gap followed closely by Adelaide Island and Elephant Island (Fig. 3). South WAP and South Shetland Islands regions had similar median transit times while transit times to the North WAP were the lowest of the 6 study regions (Fig. 3). All regions had statistically different transit times apart from the South WAP and South Shetland Islands (p = 1, p << 0.001 for all other comparisons). Transit times were pooled across DVM behavior.

**Figure 4:**
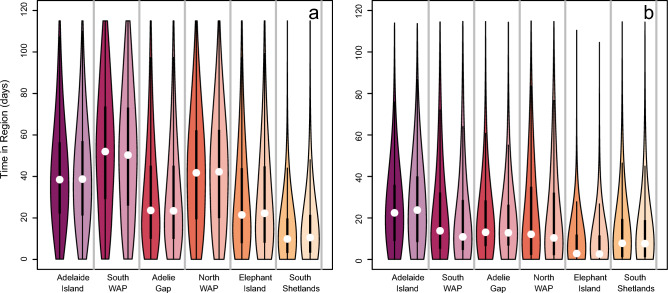
As in Fig. 3, but with the amount of time simulated krill spent in each region when they were (**a**) released within that region and (**b**) when they transited into the region from elsewhere.

We calculated the amount of time a simulated krill spent in each region separately for krill released within and outside the region of interest (Fig. 4). Simulated krill released within the South WAP spent the most time within their region of origin followed closely by the North WAP and Adelaide Island regions (Fig. 4a). The amount of time a simulated krill spent in the Elephant Island and Adélie Gap regions were similar when the krill were released there (Fig. 4a). Simulated krill released within the South Shetland Islands region spent the least amount of time there in comparison to other regions (Fig. 4a). Simulated krill spent statistically significant different amounts of time in each region when released there.

The amount of time simulated krill spent in a region where they were not released was lower than it would have been if they were released there (Fig. 4b). Simulated krill advected into the waters south of Adelaide Island spent the longest there out of all the regions (Fig. 4b). Simulated krill advected into the South WAP, Adélie Gap, and North WAP spent similar amounts of time in these regions (Fig. 4b). Krill advected into the Elephant Island and South Shetland regions spent the least amount of time there (Fig. 4b). The amount of time krill spent in each region differed significantly across all regions.

Similar to transport pathways of krill and krill transit times, the presence of DVM did not impact the time simulated krill spent in each of the study regions, regardless of if the krill started in the region, DVM behavior, or simulation year (Fig. 4, Figs. S10–11).

### Features driving connectivity

**Figure 5:**
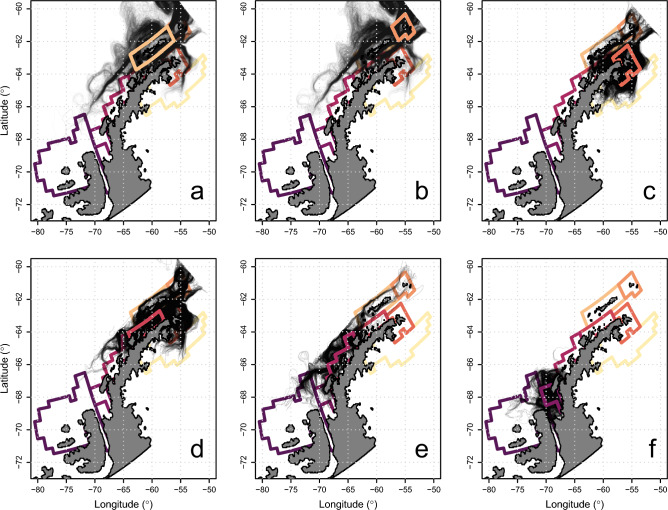
Spaghetti plots illustrating major connectivity pathways for simulated krill transported to the six study regions: South Shetland Islands (**a**), Elephant Island (**b**), North WAP (**c**), Adélie Gap (**d**), South WAP (**e**), and Adelaide Island (**f**).

**Figure 6:**
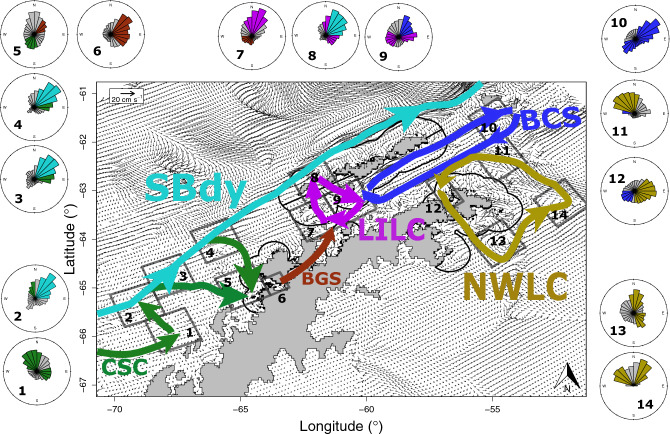
Mean current velocities and directions in the study region illustrating persistent current features. Every 25th vector is plotted. Current velocities and directions were averaged across the 4 simulated seasons during the chick-rearing period (December–March) and over the top 150 m of the water column. Arrows illustrate major current systems that play roles in facilitating or preventing krill population connectivity. Arrows size is not indicative of flow strength or relative importance. Black shapes illustrate the Voluntary Restricted Zones (VRZs) established by the Association of Responsible Krill Harvesting Companies. Differences in VRZs along the coastline reflect differences between modeled coastlines and observed coastlines. Numbered regions correspond to numbered rose plots around in the plot margins. Rose plots illustrate the distribution of current bearings from every grid point within the corresponding region in 30 degree bins. Bar heights indicate the relative frequency of that bearing and color corresponds to the current systems labeled on the main figure. Bearings illustrate the direction water was moving during the chick-rearing period at 6 depths (10, 25, 50, 75, 100, 150 m) across 4 simulated seasons.

We used the pathways of krill that interacted with our study regions (Fig. 5) to identify 6 oceanic pathways in the model that promote or inhibit connectivity between regions of the WAP: the North WAP Loop Current (NWLC), the Southern Boundary of the ACC (SBdy), Low Island Loop Current (LILC), Bransfield Current System (BCS), Bismarck and Gerlache Straits (BGS), and Cross Shelf Currents (CSC). Across season average currents (Fig. 6, Figs. S12–13, Movie S1) illustrated consistent features in the coastal ocean that drive patterns of connectivity among the regions examined here. Rose plots were used to illustrate the distribution of current directions at the intersections of these features in the chick rearing period in the top 150 m across simulated model years (Fig. 6 insets). These features are highlighted in Fig. 6.

#### North WAP Loop Current (NWLC)

The NWLC helped retain simulated krill within the North WAP region, and facilitated transport of krill to the South Shetlands, Elephant Island, and Adélie Gap (Fig. 5a–d). The NWLC consisted of the CC moving out of the Weddell and around the tip of the Peninsula on the north and east (Fig. 6 [insets 11, 13–14]). A ~ 20 cm s^−1^ current moved water to the southeast between the D’Urville and Joinville Islands and the tip of the peninsula completed the loop to the east of James Ross Island (Fig. 6 [inset 12; S12–13]; Movie S1). The northward component of this current system was the most variable component across the seasons (Fig. 6 [insets 13 and 14]; Movie S1). While the direction of this feature was consistent (Fig. 6 [insets 13 and 14]), the speed varied across daily averages (Movie S1).

#### Southern Boundary of the ACC (SBdy)

Some simulated krill from the regions south of the Adélie Gap (Bellingshausen Sea, Adelaide Island, and South WAP) were advected along the continental shelf via the SBdy (Fig. 5a,b,d,e). This feature facilitated the transport of simulated krill from these southmost regions to the South Shetland and Elephant Island regions (Fig. 5a,b) and moved rapidly (> 20 cm s^−1^) and consistently along the continental shelf break (Fig. 6 [insets 2–4, 8]; Movie S1). This feature also intersected with the LILC in the Boyd Strait (Fig. 6 [inset 8]) and dominated the northeasterly component of the flow in that region. Some simulated krill advected through the Boyd Strait from the south via the LILC were transported by the SBdy along the north shore of the South Shetland Islands (see below; Fig. 5a,b).

#### Low Island Loop Current (LILC)

The LILC consisted of 3 currents: (1) a northward current around Low Island; (2) a southeasterly current along the coast of Snow and Deception Islands; and (3) a southwesterly return flow from approximately Tower Island to Hoseason Island (Fig. 6 [insets 7–9]; Movie S1). This current had consistent speeds of approximately 20 cm s^−1^ (Fig. 6 [inset 7]; Movie S1). The northward component of this flow acted as a major barrier for simulated krill entering the Adélie Gap and points north from southern regions, and for simulated krill entering points south of the Adélie Gap from northern regions (Fig. 5c,e). The southwesterly component of this current system was the most variable of the three components of the system and as a result is more prevalent in daily averages in comparison to the season averaged currents (Fig. 6 [insets 7, 9]; Movie S1). The LILC also helped retain simulated krill within the Adélie Gap (Fig. 5d). When this feature intersected with the SBdy to the north, krill could be exchanged between the two features (Fig. 5a–e). While the SBdy dominated flows in the Boyd Strait (Fig. 6 [inset 8]), the LILC was able to keep some krill inshore, especially if they were already inshore (Fig. 5b,c). Krill already in the SBdy were more likely to stay there whereas krill advected northward by the LILC had a chance of getting pushed into the SBdy (Fig. 5a–c).

#### Bransfield Current System (BCS)

The BCS consisted of (1) a northeasterly current along the south coast of the South Shetland Islands toward Elephant Island; (2) a southward current from Elephant Island towards the tip of the peninsula; (3) the CC moving out of the Weddell and to the southwest along the peninsula; and (4) a northward current between Tower and the South Shetland Islands (Fig. 6 [insets 9–12]; Movie S1). These currents moved rapidly (~ 20 cm s^−1^) and were relatively consistent (Fig. 6 [insets 9–12]; Movie S1). The southward component of the BSC that returned krill around Elephant Island to the Peninsula was the least consistent component of this flow, with speeds varying widely (Movie S1). The BCS facilitated most of the transport from the South Shetland Islands to Elephant Island and helped retain simulated krill around the Adélie Gap (Fig. 5a–d).

#### Bismarck and Gerlache Straits (BGS)

The BGS between Anvers Island and the Antarctic Continent served as the primary feature connecting the South WAP and Adélie Gap regions (Fig. 5d). Water moved rapidly through this tight channel (~ 20 cm s^−1^; Fig. 6, Movie S1). While across-season averaged currents illustrate this feature moving water, and therefore krill, towards the continent, daily currents show the net northeasterly movement of water through this region (Fig. 6 [inset 6]; Movie S1).

#### Cross Shelf Currents (CSC)

The CSC consisted of persistent currents moving from the continental shelf inshore (Fig. 6 [insets 1–5]; Movie S1). These shoreward currents had relatively consistent speeds (~ 10 cm s^−1^) across the depths considered (Fig. 6 [insets 1–5]; Figs. S12–13). Unlike other features described here, the CSC refers to a set of three similar current systems along the continental shelf of the WAP. These currents facilitated the transport of simulated krill from the Bellingshausen Sea and Adelaide Island regions into the South WAP region, and likely helped retain simulated krill within the South WAP (Fig. 5e). The offshore components of the CSC also moved krill into the SBdy (Fig. 5a,b,d,e; Fig. 6 [inset 2]), which was the dominant current when the two features intersected (Fig. 6 [inset 2–4]). Similar current systems helped retain krill around Adelaide Island (Fig. 5e).

### Penguins impacted by persistent features

We determined the total number of penguins adjacent to these persistent ocean features using a Bayesian population dynamics model^85^, which allows us to integrate all available census data to predict the current number of breeding penguins at each location (Tables 1, 2; Fig. 1). We then identified the persistent current features that serve as a source of krill to each of the study regions (Fig. 6). The South WAP and Adelaide Island regions were primarily supported by the CSC (Fig. 6). While Adelaide Island was dominated by several small and one large Adélie penguin colony (Fig. 1), all three *Pygoscelis* penguin species were present in several small colonies within the South WAP (Table 1). Gentoos made up the majority of *Pygoscelis* penguins in the South WAP (Table 1). Similarly, the North WAP region received krill primarily from a single current system—the NWLC (Fig. 6; Table 1). This region contains the most penguins of any region, containing over 1 million Adélie penguins (Table 1).

**Table 1:** Number of nests (and number of colonies) within the study regions on the West Antarctic Peninsula (WAP). Penguin population data are from Mapping Application for Penguin Populations and Projected Dynamics (MAPPPD) predictions.

Penguin species	Region					
	Elephant Island	South Shetland Islands	North WAP	Adélie Gap	South WAP	Adelaide Island
*Adélie*	1666 (2)	7731 (9)	1,002,209 (29)	0 (0)	13,361 (35)	82,217 (8)
*Chinstrap*	227,189 (53)	563,916 (123)	20,568 (5)	36,355 (56)	3727 (12)	0 (0)
*Gentoo*	6318 (7)	71,378 (21)	10,810 (15)	34,797 (15)	34,797 (30)	0 (0)
Total	235,173 (62)	643,025 (153)	1,033,587 (49)	71,152 (83)	51,363 (77)	82,217 (8)

**Table 2:** Spearman’s rank correlation, and associated 95% confidence intervals, between penguin abundances and calculated connectivity metrics within each region illustrated in Figs. 3 and 4.

Penguin species	Connectivity metric		
	Transit time	Time in region (released within)	Time in region (released outside)
*Adélie*	− 0.4 (− 0.9 to 0.7)	0.7 (− 0.5 to 1)	0.7 (− 0.5 to 1.0)
*Chinstrap*	0.5 (− 0.6 to 1.0)	− 0.9 (− 1.0 to − 0.1)	− 0.6 (− 1.0 to 0.6)
*Gentoo*	0.2 (− 0.8 to 0.9)	0.1 (− 0.9 to 0.9)	0.4 (− 0.7 to 0.9)
All	− 0.4 (− 0.9 to 0.6)	− 0.3 (− 0.9 to 0.7)	− 0.2 (− 1.0 to 0.9)

The Adélie Gap received krill via three persistent current features identified here: the BGS, LILC, and BCS (Fig. 6). While there are nearly four times the number of chinstrap colonies than gentoo colonies present in this region, the numbers of gentoos and chinstraps were nearly equal (Table 1). The South Shetland Islands also receive krill from three current features: the LILC, BCS, and ACC (Fig. 6). Chinstrap penguins dominate this region in both numbers of individuals and colonies (Table 1). Elephant Island also receives krill via the BCS and SBdy (Fig. 6). Similar to the South Shetland Islands, chinstrap penguins predominate (Table 1).

Since connectivity metrics did not differ when simulated krill performed DVM or were passive in the horizontal, metrics with and without DVM were pooled for correlation to penguin populations within our study regions (Table 2). Penguin populations and our connectivity metrics were not significantly correlated (Table 2).

## Discussion

*Pygoscelis* penguins primarily consume krill during the austral summer^33,43,86^. Krill distributions along the WAP are spatially and temporally heterogeneous^1–6,31^ and are facing increasing pressures from a growing krill fishery^17^. Therefore, the connectivity of krill could not only play an important role in supplying necessary resources to penguin colonies, but also could be the key to their sustainable management. Previous modeling work has focused either on krill from the Weddell or the Bellingshausen Seas. Here, we used an ocean circulation model to determine how simulated krill are connected across coastal regions along the WAP, including both the Weddell and Bellingshausen Seas as possible sources. We hypothesized that krill originating from the Weddell and Bellingshausen Seas would supply different penguin populations, the addition of DVM to simulated krill would alter krill transport pathways, and that presence and the timing of this connectivity around the Peninsula may play a role in penguin population dynamics in the region.

Here, we found that the Bellingshausen Sea served as the primary source of krill for regions south of the Adélie Gap and the Weddell Sea provided krill to regions north of this region, supporting our hypothesis. Connectivity between these regions is limited by a northward current around Low Island within the Adélie Gap. This current, like many along the WAP, appears to be bathymetrically driven, following the contours of Boyd Strait between Low Island and the South Shetland Islands (Fig. 1)^27^. This current likely acts as a boundary between the Bransfield Strait and points south on the Peninsula, which have very different water column structures and water mass properties^87^.

These patterns of connectivity between regions are remarkably consistent, with low variability across four different austral summers, and most are associated with bathymetric features. The CSC, for example, are driven by troughs and canyons crossing the continental shelf and the BCS follows bathymetric contours in the region. Persistent features were not found in areas on the continental shelf without strong bathymetry changes, illustrating the importance of bathymetry, and the resulting bathymetric steering of ocean currents in this region^88–90^.

A majority of the persistent current features described here that drive krill connectivity along the WAP, including the ACC^27,91^, CSC^88–90^, BGS^27,92^, BCS^28,29,92–94^, and LILC^91–93^, have been observed along the WAP. Both the LILC and BCS have their components described in detail but are not often considered closed loop systems as we have described them here. Entrainment of simulated krill by both these features is present, albeit not persistently in our observations. Therefore, more observations of these systems are necessary to determine if these features persist as closed loop systems or are simply connecting different current systems.

While the component of the NWLC associated with the CC has been observed previously^29,91,95^, observations suggest that flow between the D’Urville and Joinville Islands and the tip of the WAP is northward, rather than southward^94,95^. Current distributions in this region suggest that northward flow is possible, however, this region is dominated by south and easterly flows. Furthermore, observed local water mass properties suggest that northward currents through this region is unlikely^96^. Therefore, additional observations are necessary to determine if the southward component of the NWLC is present and persistent feature during the austral summer.

Krill entering the North WAP had the shortest transit times and some of the longest entrainment times in comparisons to other study regions. Krill spent similar amounts of time in the South WAP and Adelaide Island regions in comparison to the North WAP but the transit times to these regions were longer. This is likely because the Bellingshausen Sea serves as the primary source of larval and juvenile krill to these regions, and is farther away from these study regions, whereas the North WAP and Weddell Sea regions were adjacent to each other. In addition, our sources of krill in the Bellingshausen and Weddell Seas may be differentially impacted by changes to the environment observed over the last several decades. The Weddell Sea may serve as a krill sanctuary due to the extent and persistence of sea ice in the region, whereas sea ice—a critical overwintering habitat for krill^9–12^—is declining in the Bellingshausen^97–99^. Changing krill stocks and distributions as a result of climate change^1,2,74^, albeit debated^73,100^, have been linked to penguin population declines^16,101^, changes in diet compositions in gentoos^102^, and reproductive success of other krill predators such as the Antarctic fur seal (*Arctocephalus gazella*)^103^ throughout the WAP, suggesting that krill availability may be declining to predators.

These transport mechanisms, and resulting connectivity metrics, were insensitive to the presence or absence of DVM behaviors. Though we had hypothesized a bigger role for DVM because previous modeling studies in this region had shown that DVM increased the retention of resources locally^61^, our results are consistent with prior work illustrating DVM had only a small (< 10%) effect on transport pathways for simulated krill larvae released off the continental shelf^22^. It is likely that we did not observe the influence of DVM due the broad spatial scope of our study region, which included both rapidly-moving bathymetry-driven currents such as the ACC and slower continental shelf currents. In addition, currents on the WAP shelf are relatively uniform in the vertical, especially over the scales considered here^93^. This is especially true down to the pycnocline, which separates surface currents from deep currents in this region^52^. Summertime pycnoclines range from ~ 30 to 80 m on the WAP shelf^52,87,104^, meaning that simulated krill performing three of our five DVM behaviors would not leave the rapidly-moving surface layer. This iteration of ROMS has been shown to underestimate vertical shear on the shelf, so even if krill migrated below the pycnocline, it may not have a significant impact on these simulations^52,87^. Furthermore, we assumed that krill were only actively swimming in the vertical and were passive in the horizontal, primarily due to a lack of a krill movement model on the scale of our model. Previous studies in other physical ocean models have illustrated the importance of directed horizontal movement in both the presence and absence of DVM on small and large spatial scales^6,105^. Therefore, building a more realistic krill movement model that will allow us to better understand the role of advection on even smaller spatial scales represents a future research priority.

None of the correlations between connectivity and penguin abundance were statistically significant, which we attribute to the low statistical power resulting from the relatively small number of study regions. This reflects an inherent statistical challenge without easy solutions, in that penguins forage over a large region but these large regions provide a relatively small number of independent samples from which to rigorously test how krill dynamics may influence penguin populations. A more mechanistic understanding of how these metrics influence penguin abundance will require (1) expanded tagging of foraging penguins across all three *Pygoscelis* species in concert with methods, such as underwater cameras, to establish prey choice as it relates to prey availability, (2) expanded mapping of krill densities on fine spatial scales (e.g. using glider-based acoustic surveys, ship-based tows or acoustic surveys), and (3) data on commercial krill catches at finer spatial scales than are currently available to the public.

Another explanation for the non-significant correlations may be that the larger colonies north of the Adélie Gap could persist due to transport of other prey species such as Antarctic silverfish (*Pleuragramma antarcticum*)^106–109^. All life stages of the silverfish are strongly dependent on sea-ice extent^110^, and juvenile and larval silverfish would be transported by the same persistent current systems described here. Therefore, the Weddell Sea may also serve as an important refuge for silverfish. Previous modeling studies have illustrated that larval silverfish can be transported from the Weddell Sea to the North WAP and Adélie Gap, likely through the NWLC and BCS described here^111^. In addition, the LILC may continue to act as a barrier to transport south of the Adélie Gap^111^. Increased availability of silverfish via the persistent current features described here, therefore, may be an additional driver of penguin population dynamics north of the Adélie Gap. Silverfish are noticeably absent from penguin diets south of the WAP. However, the presence of smaller persistent current features may retain enough krill near penguin colonies to allow them to persist^51,106,112^.

While our connectivity metrics did not significantly correlate with penguin colony sizes in our study regions, patterns in our metrics align with previous hypotheses that prey could become limiting within the BCS and around the Adélie Gap. The Bransfield Strait between the South Shetland Islands and the coast of the WAP is a hotspot for krill recruitment and a plethora of krill predators, including the pygoscelid penguins that forage in this region^24^. This suggests that prey resources should be plentiful enough to facilitate successful penguin foraging and colony establishment in the Adélie Gap. Trathan et al.^19^ hypothesized that krill could become limited within this region in low krill years and high predator and/or fishery demand. Our simulated krill had relatively longer transit times and spent less time in the Adélie Gap in comparison to other regions, supporting the hypothesis that krill could become limiting within this system.

In addition, long transit times may prevent Adélie penguins specifically from forming colonies within this region due to their breeding phenology. Adélies breed the earliest among the *Pygoscelis* penguins^42^. If krill are not abundant within the Adélie Gap when they return to their colonies, they could find themselves in a resource-limiting environment that is not conducive to the kind of spatially constrained foraging required by egg incubation. While the coastal region immediately adjacent to the Adélie Gap may serve as a krill recruitment hotspot for juvenile krill, our results indicate that these krill recruits would be quickly advected out of the Adélie Gap and into the South Shetland Islands and Elephant Island regions via the BCS. Furthermore, Adélie penguins tend to consume krill larger than 30 mm^113^, whereas year one recruits tend to be anywhere from 20 to 30 mm long^114^. Therefore, even if this region is a krill recruitment hotspot, the krill retained within the region may be too small. Gentoos and chinstraps may be able to persist due to higher diet plasticity^115,116^. More research to determine predator demands and prey availability within this region are critical to determining if resource limitation is responsible for the formation of the Adélie Gap and how the krill fishery should be sustainability managed.

Our results suggest that while the voluntary no-take regions established by ARK may protect the areas directly around penguin colonies, additional measures may be required to accommodate the interconnectedness identified by our model, particularly as the Bellingshausen and Weddell Seas serve as critical sources of krill to multiple regions of the Peninsula that come together in the Adélie Gap through the LILC. Our results serve as a reminder that management of krill at smaller spatial scales, as suggested by previous studies^18,20,21,35,49^, will need to accommodate the connectivity imposed by the region’s hydrography. Specifically, our results highlight that marine protected areas (MPAs) upstream of the South and North WAP regions would protect critical prey resources for predators. The protection of upstream krill represents an important precautionary measure to avoid the irreversible damage that human activities could cause to the WAP food web, particularly given our improved understanding of krill transport and connectivity.

## Supplementary Information

Supplementary Information 1.

Supplementary Information 2.

## Data Availability

Bounding boxes for the regions used in this study, indexes used to subset simulated krill released within each region, and the code used to conduct connectivity calculations are available on GitHub (https://github.com/klgallagher/connectivity). Simulated krill trajectories with and without DVM, and current velocity and direction data are available through the United States Antarctic Program Data Center (https://www.usap-dc.org/view/project/p0010349).
