# Supplementary figures and images for: Quantifying Antarctic krill connectivity across the West Antarctic Peninsula and its role in large-scale Pygoscelis penguin population dynamics
Source: Sci Rep. 2023 Jul 26;13:12072. doi: 10.1038/s41598-023-39105-6 (PMC10372022; doi:10.1038/s41598-023-39105-6)

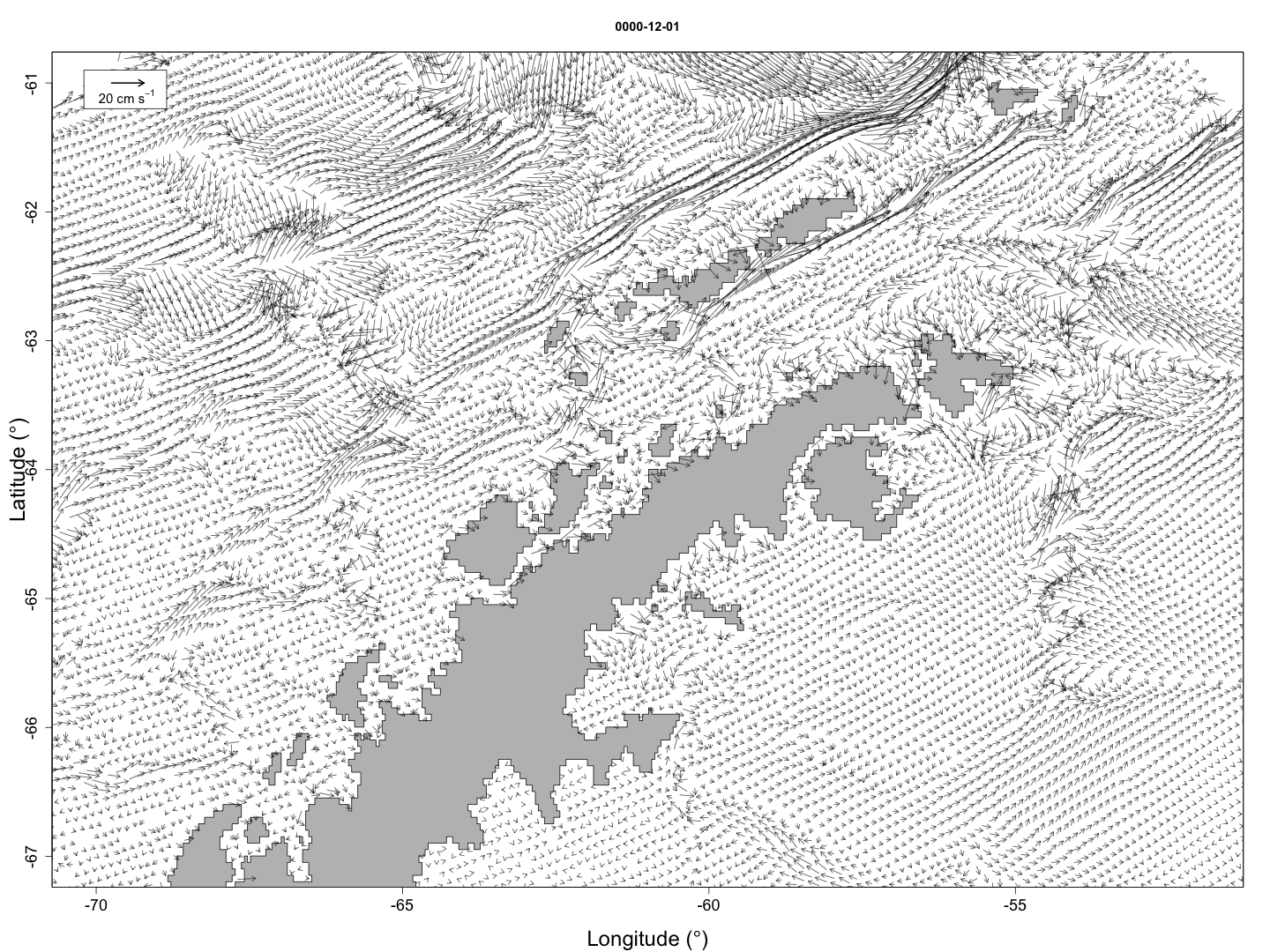

Supplement: Supplementary file 1 — Supplementary Information 1. [file 41598_2023_39105_MOESM1_ESM.gif]
