# Supplementary material for: Quantifying Antarctic krill connectivity across the West Antarctic Peninsula and its role in large-scale Pygoscelis penguin population dynamics
Source: Sci Rep. 2023 Jul 26;13:12072. doi: 10.1038/s41598-023-39105-6 (PMC10372022; doi:10.1038/s41598-023-39105-6)
Supplement: Supplementary file 2 — Supplementary Information 2. [file 41598_2023_39105_MOESM2_ESM.pdf]

Supplementary Material For

Quantifying Antarctic krill connectivity across the West Antarctic Peninsula and its role in large-scale *Pygoscelis* penguin population dynamics

Katherine L. Gallagher[1*], Michael S. Dinniman[2], Heather J. Lynch[1]

1. Institute for Advanced Computational Sciences, Stony Brook University, Stony Brook NY 11794, USA
2. Department of Ocean and Earth Sciences, Old Dominion University, Norfolk VA 23529, USA

Supplementary Text A – Model Simulation Details

The iteration of the Regional Ocean Modeling System (ROMS) for the West Antarctic Peninsula (WAP) was run on the Ookami High Performance Computing cluster at Stony Brook University and compiled using Fujitsu compilers. This is one of the few instances of ROMS compiled with Fujitsu to our knowledge and produced identical results to previous simulations compiled with GNU compilers. Simulations were run across 32 nodes, utilizing 18 of the 24 cores of each node to provide enough memory for the model. Each simulation had a total of 11,156 simulated krill released in each of 16 release events. This gave a total of 178,496 simulated krill released in each simulation. Simulated krill were subset to the study regions and simulated krill released under ice shelves were not considered (Figure S1). The 20 simulations (4 seasons x 5 DVM behaviors) required for this project each took an average of 2.4 days to complete on Ookami, for a total wall time of 48 days. All simulations were run continuously (no restarts). Simulations without DVM behaviors were also conducted with simulated krill released in the same locations. For these simulations, only float trajectory data was saved to save computing time. These runs took an average of 1.7 days, for an additional wall time of 34 days.

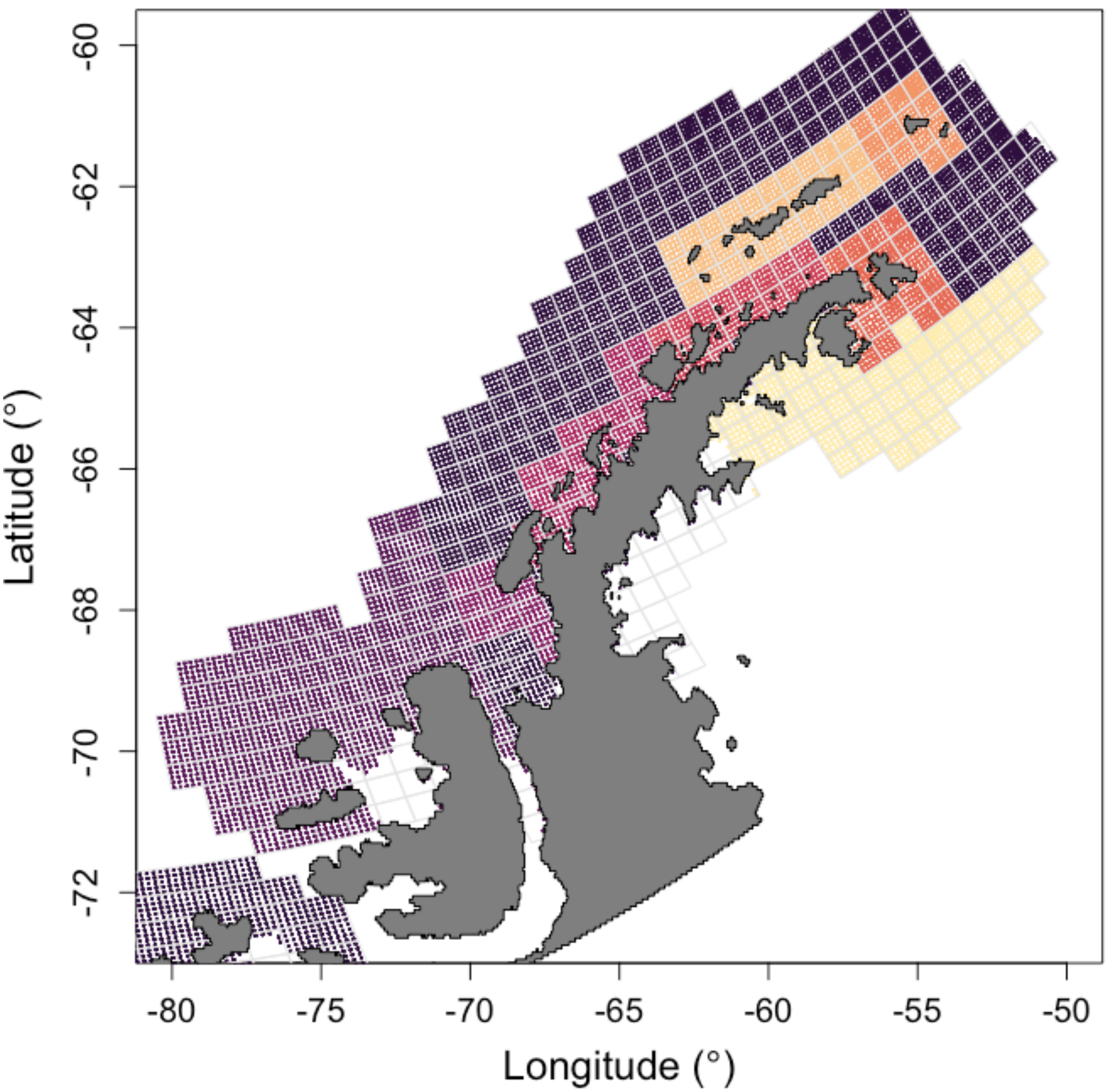

Figure S1. The study region with simulated krill release locations. Points are colored by region of origin. Grey boxes outline the 40 km^2 grid used to assist in region formation.

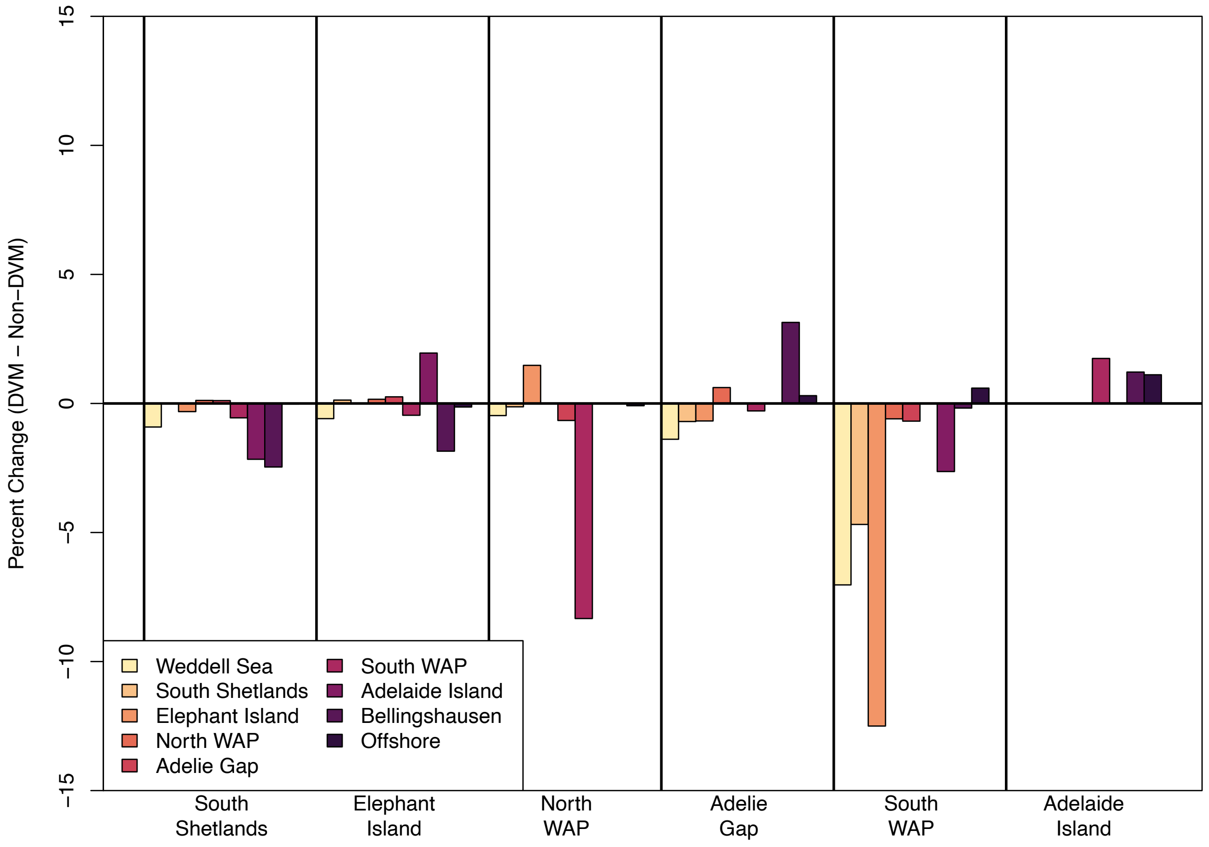

Figure S2. Percent differences in the number of simulated krill that entered the study regions averaged across model years and diel vertical migration (DVM) behaviors. Color indicates the region in which the krill was released. Positive values indicate that more krill entered the region in the presence of DVM, while negative values indicate that the number of krill that entered the region from each source region was greater when krill did not perform DVM.

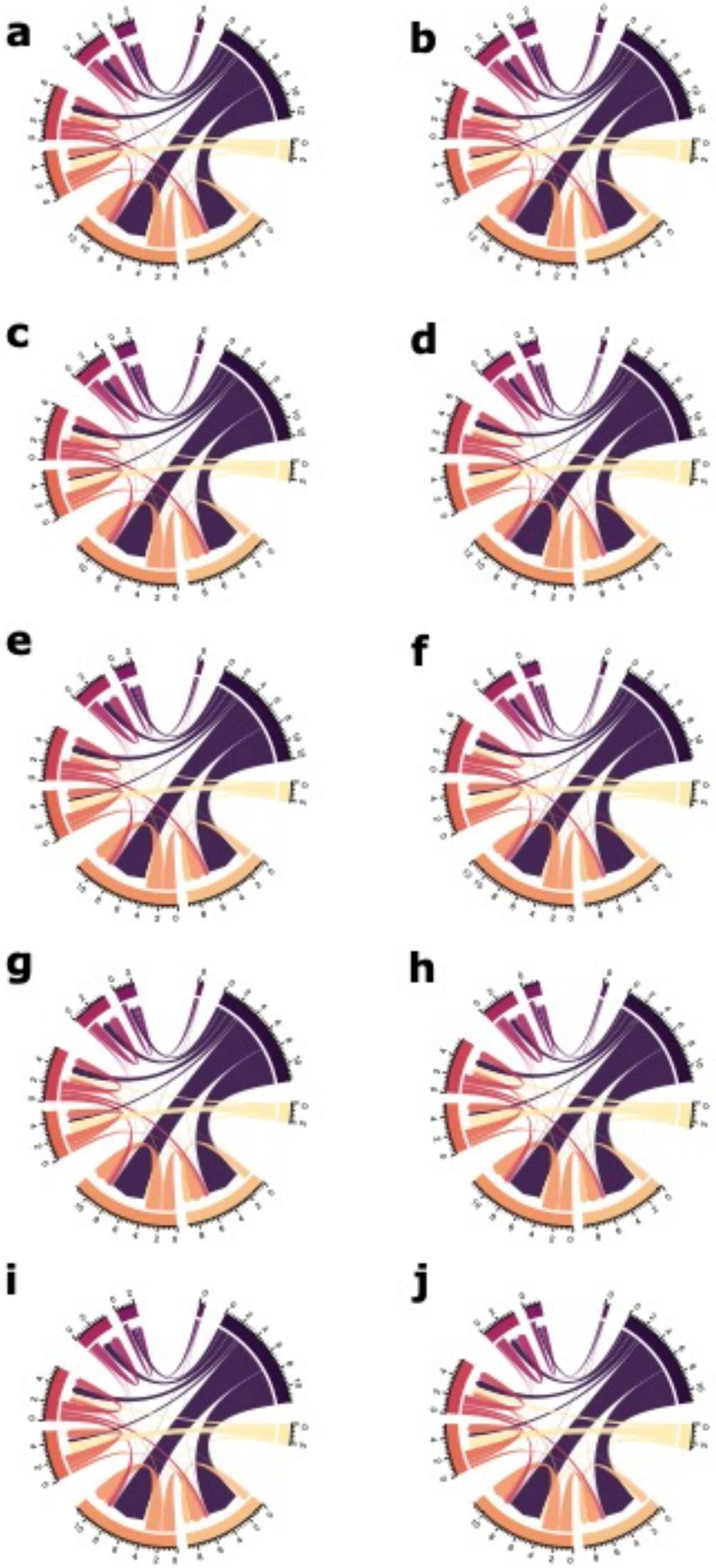
a
b
c
d
e
f
g
h
i
j

Figure S3. Chord diagrams illustrating interannual averages of the number of krill that entered each of the study regions from source regions (Bellingshausen, Offshore, and the Weddell Sea) or other study regions (all other regions) for simulated krill released within the 2008 season across vertical migration behaviors. Simulated krill performed diel vertical migration down to (a, c, e, g, i) or were released without DVM behavior (b, d, f, h, j) to 25 (a-b), 50 (c-d), 75 (e-f), 100 (g-h), and 150 (i-j) m. Colors represent each region and are the same as Figure 2 in the main text.

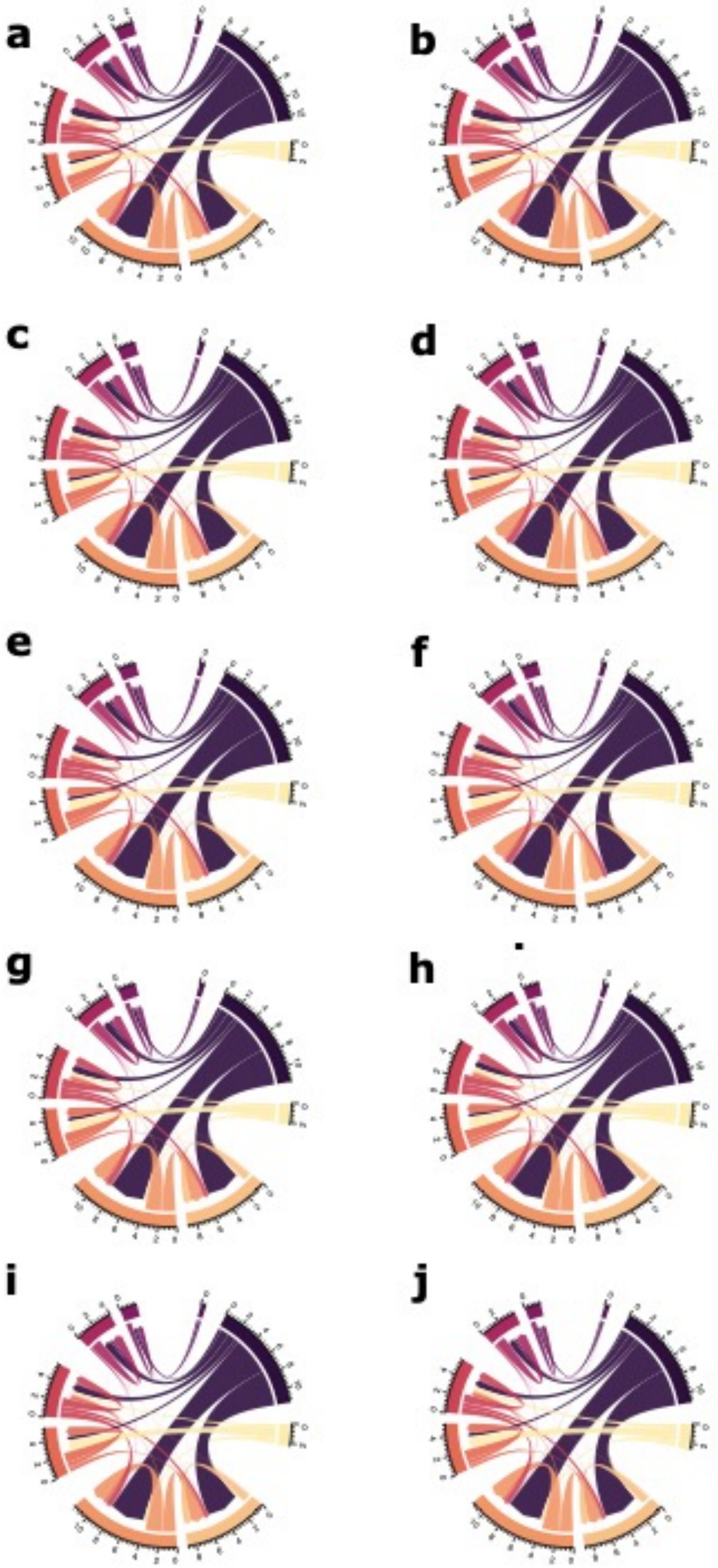
a
b
c
d
e
f
g
h
i
j

Figure S4. As in Figure S3, but with simulated krill released in the 2009 season.

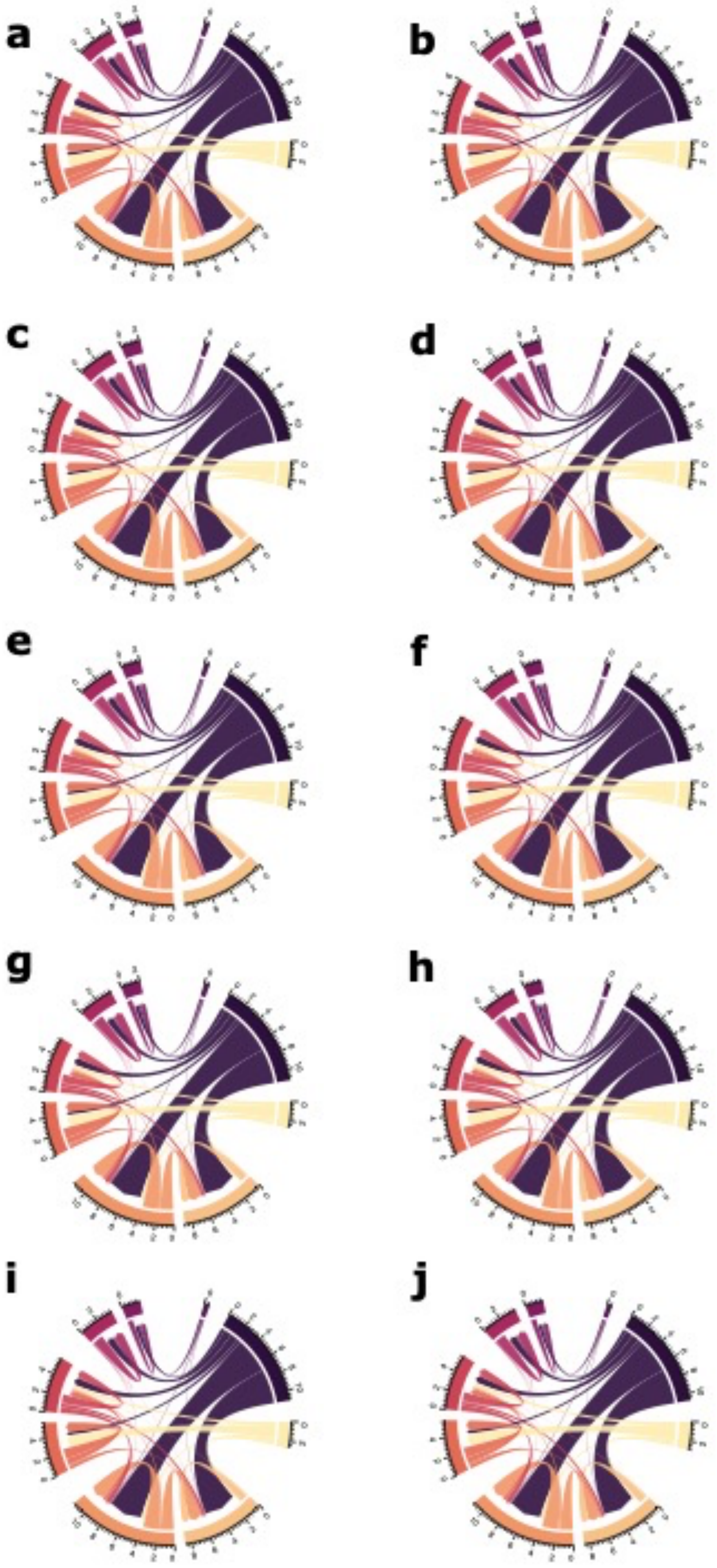
a
b
c
d
e
f
g
h
i
j

Figure S5. As in Figure S3, but with simulated krill released in the 2018 season.

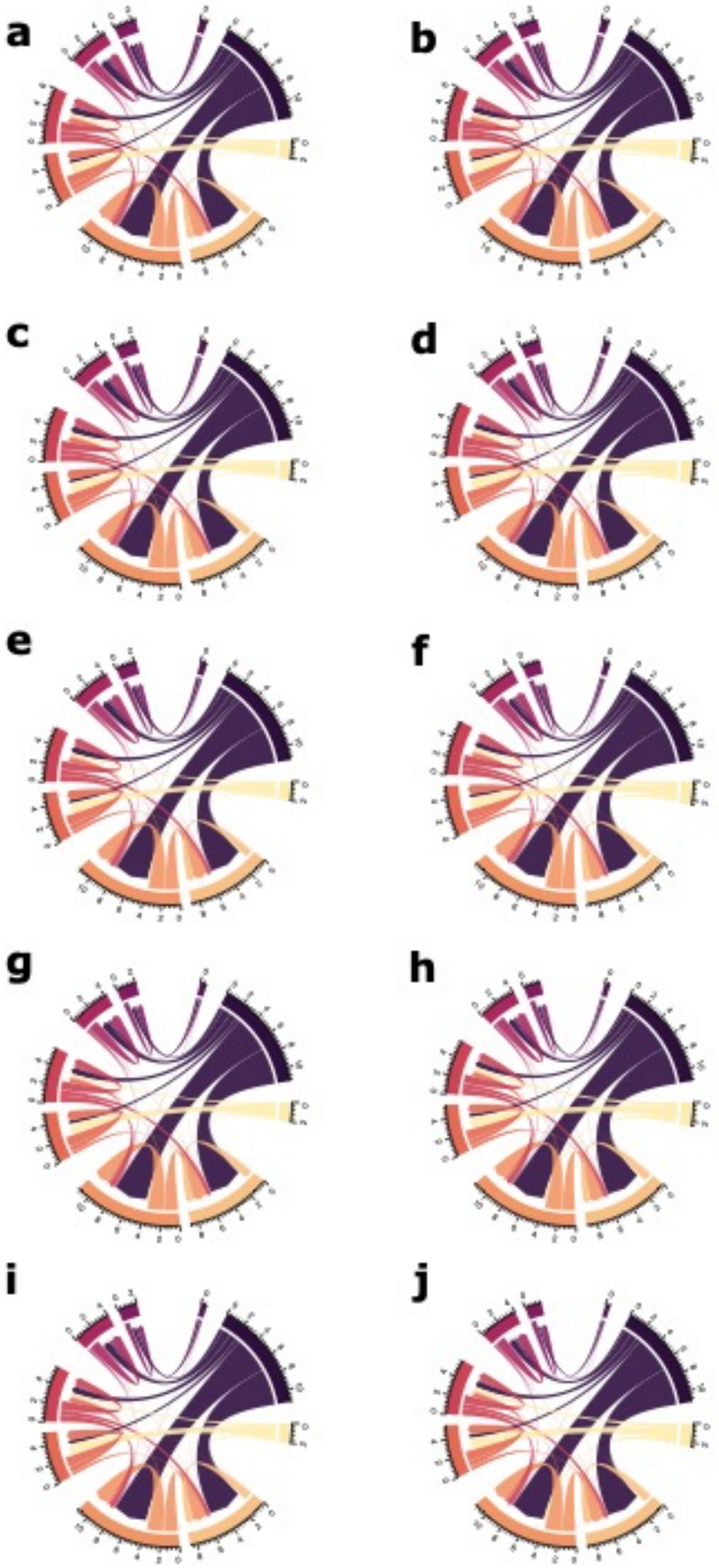
a
b
c
d
e
f
g
h
i
j

Figure S6. As in Figure S3, but with simulated krill released in the 2019 season.

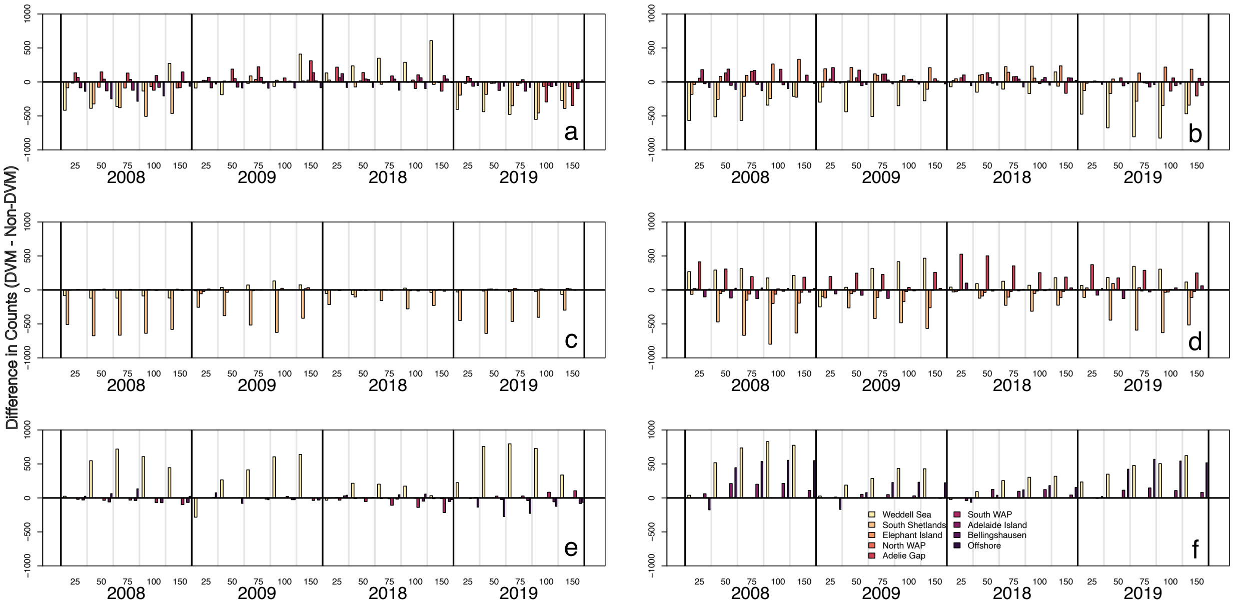

Figure S7. Differences in the number of simulated krill that entered each study region, separated by model year and diel vertical migration behavior. Positive values indicate that more krill entered the region in the presence of DVM, while negative values indicate that the number of krill that entered the region from each source region was greater when krill did not perform DVM. Panels represent the following regions: South Shetland Islands (a), Elephant Island (b), North West Antarctic Peninsula (WAP) (c), Adelie Gap (d), South WAP (e), and Adelaide Island (f).

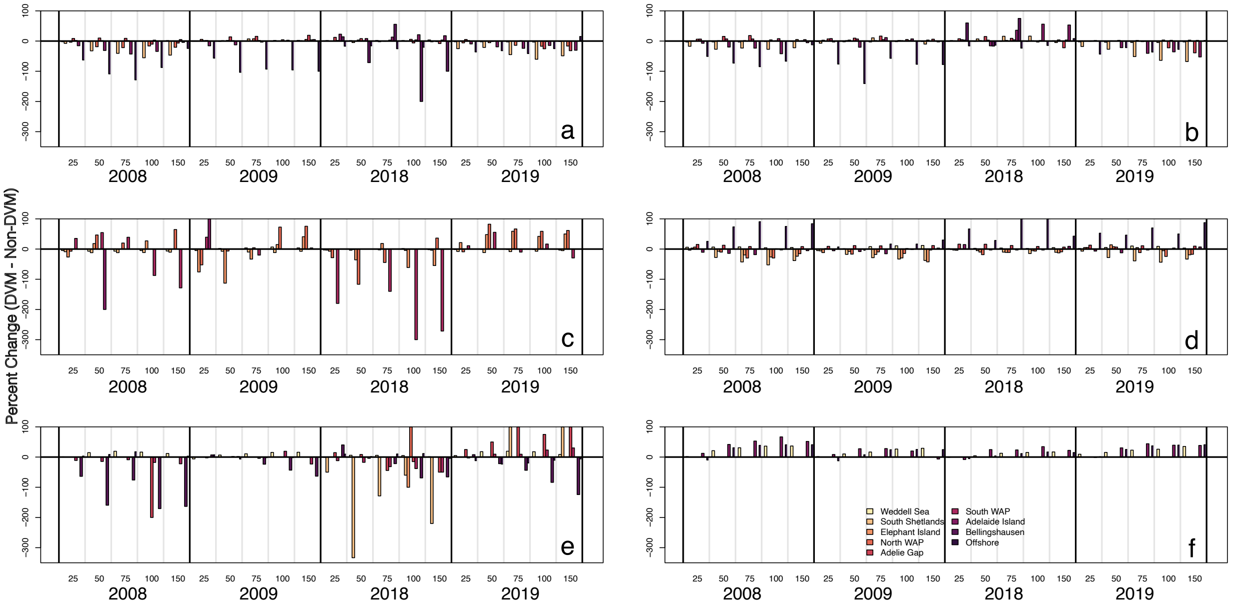

Figure S8. As in Figure S7, but with percent change in the number of simulated krill that entered each study region.

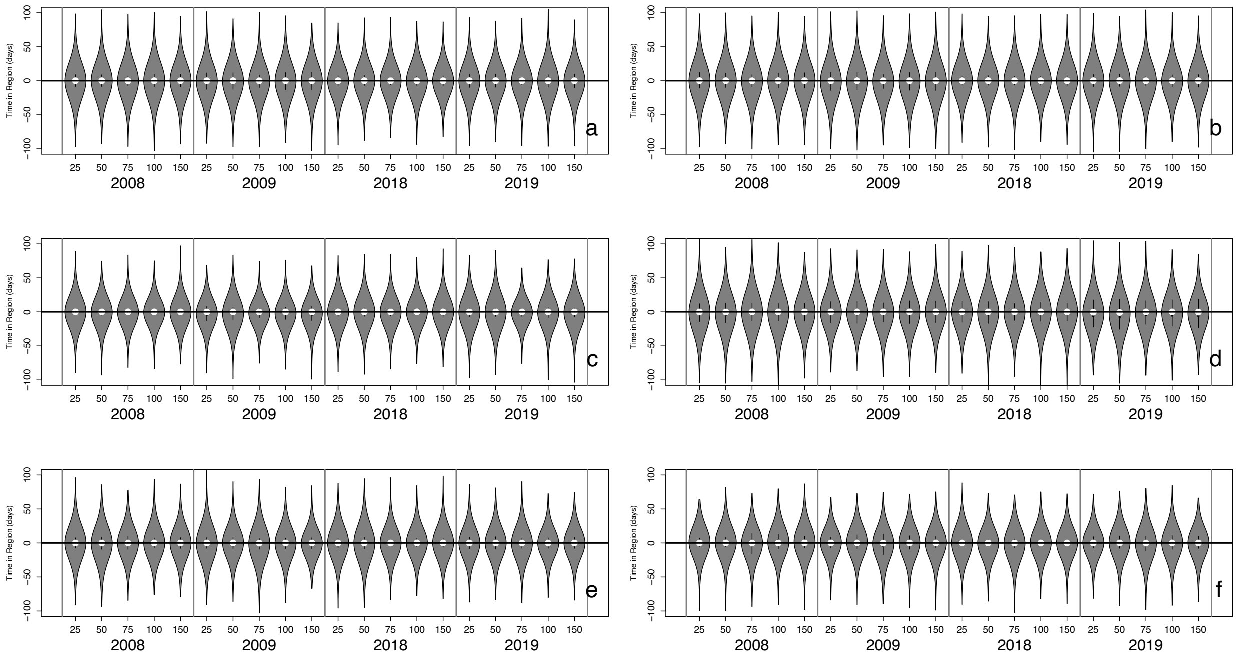

Figure S9. Violin plots illustrating the differences in transit times for simulated krill entering the study regions. White points indicate the median difference in transit time and black lines illustrate the interquartile range. Width of the bars indicate the distribution of the data, with wider bars indicating more data is present at that value. Positive values indicate that transit times to the region were longer in the presence of DVM, while negative values indicate that transit times the region were longer from each source region was greater when krill did not perform DVM. Panels represent the following regions: South Shetland Islands (a), Elephant Island (b), North West Antarctic Peninsula (WAP) (c), Adelie Gap (d), South WAP (e), and Adelaide Island (f).

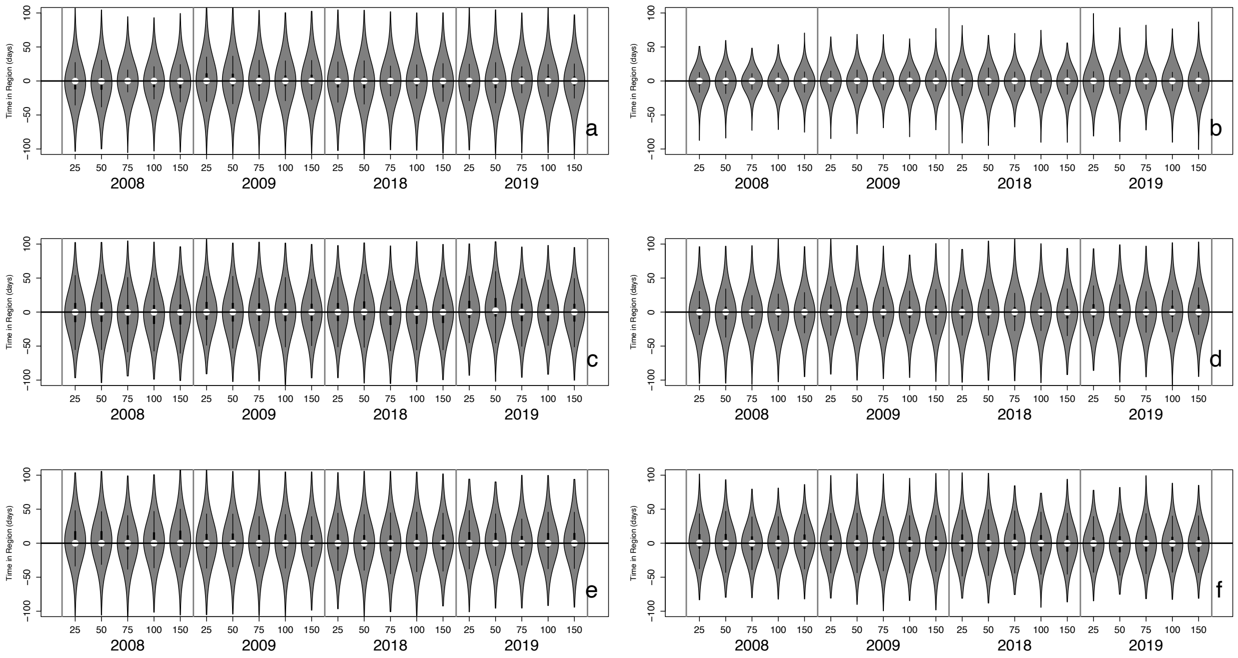

Figure S10. As in Figure S9, but illustrating differences in the number of hours spent in each region for simulated krill released in each region.

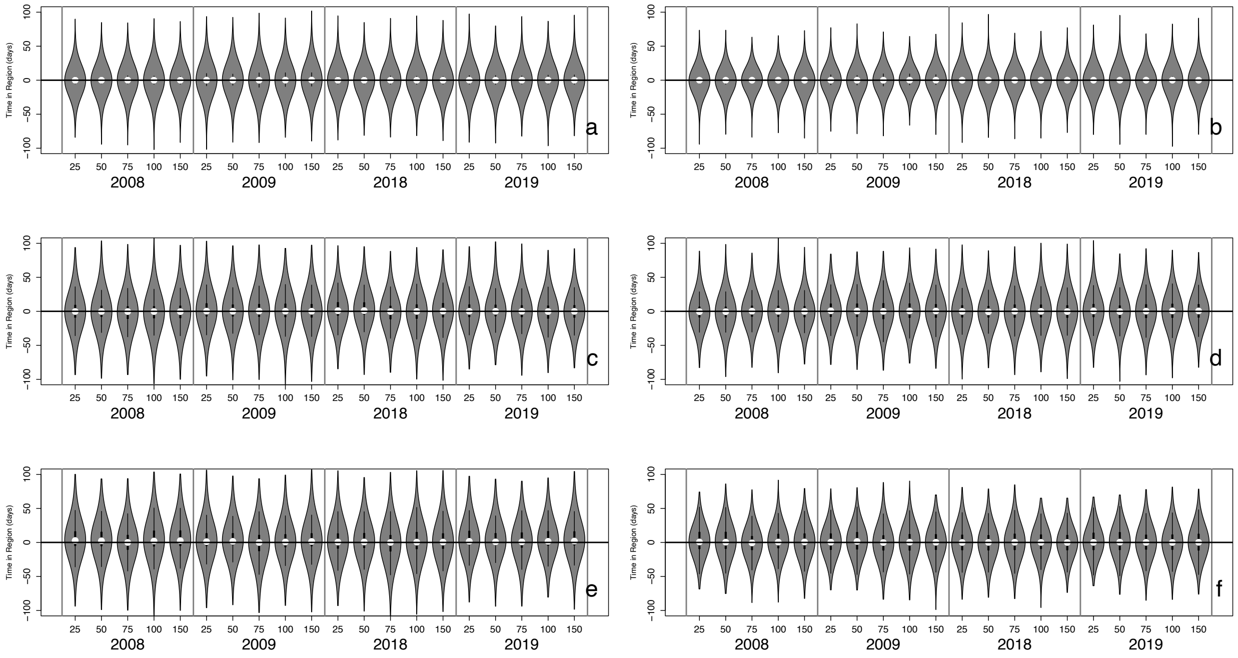

Figure S11. As in Figure S9, but illustrating difference in the number of hours spent in each region for simulated krill released outside each region.

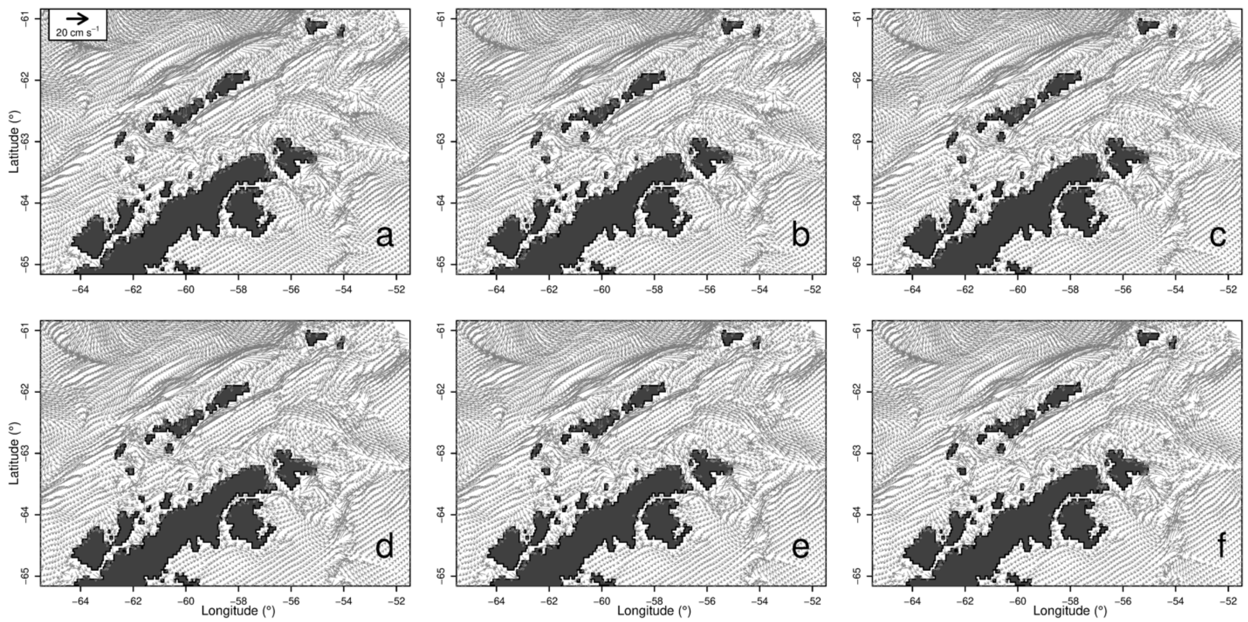

Figure S12. Mean current velocities at 10 (a), 25 (b), 50 (c), 75 (d), 100 (e), and 150 m (f) in the northern half of the study region, including the Weddell Sea, North West Antarctic Peninsula (WAP), Adelie gap, South Shetland Islands, and Elephant Islands regions. Current velocities were averaged across the 4 austral summers simulated and averages were calculated for the chick-rearing period (December – March). Every 30th vector is plotted.

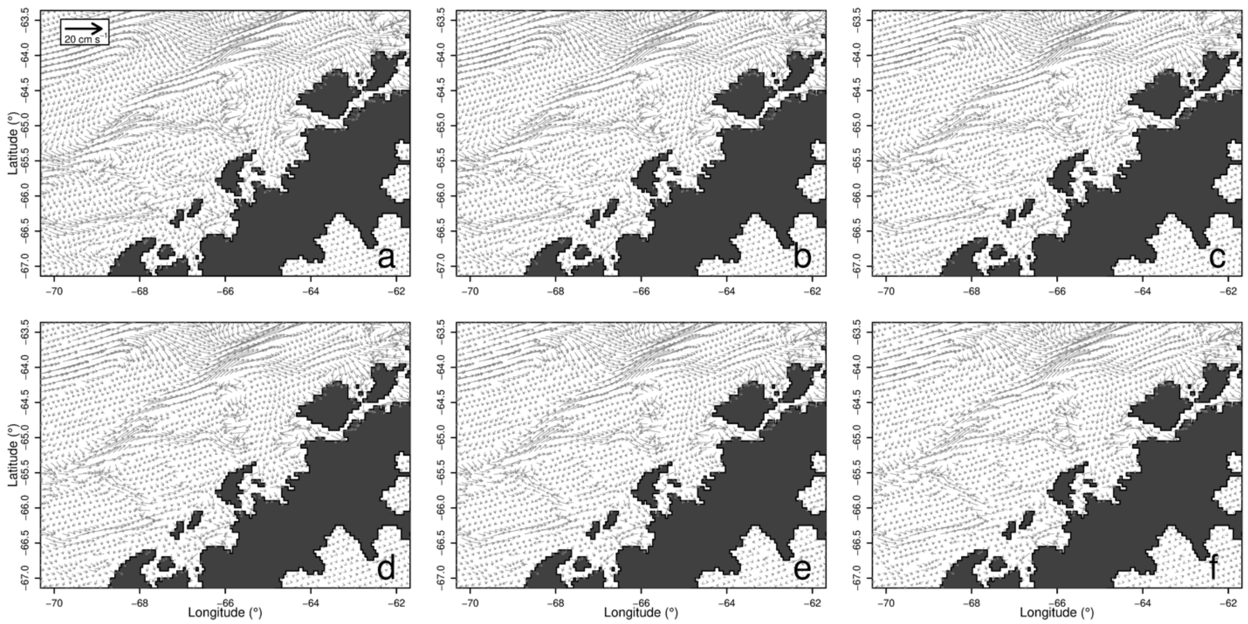

Figure S13. As in Figure S12, but for the southern half of the study region, including the South West Antarctic Peninsula (WAP) and Adelaide Island regions.

Supplemental Tables:

Table S1. Previously published krill diel vertical migration (DVM) observations along the West Antarctic Peninsula (WAP) used to inform DVM simulations.

Daytime Depths (m)	Nighttime Depths (m)	Location	Season	Citation
37 – 48	NA	Palmer Deep	Summer	Bernard et al 2017[55]
20 – 200	NA	Palmer Deep	Summer	Nardelli et al 2020[56]
30	NA	Palmer Deep	Summer	Cimino et al 2016[57]
100 – 140	20 – 30	South Georgia	Summer	Everson et al 1983[58]
250	Near surface	Marguerite Bay & WAP continental shelf	Fall – Early Winter	Zhou and Dorland 2004[59]
45 – 100	25 – 85	Northern WAP/South Shetland Islands	Summer	Godlewska and Klusek 1987[60]
200 – 400	Upper 200	Wilhelmina Bay	Fall	Espinasse et al 2012[61]
40 – 80	20 – 40	Palmer Deep	Summer	Goodrich 2018[62]
50 – 250	Upper 100	Branford Strait bays	Late spring	Kane et al 2018[63]
10	50	Palmer Deep	Summer	Hudson et al., 2022[53]

Table S2. Previously published *Pygoscelis* penguin foraging depth observations along the West Antarctic Peninsula (WAP) used to inform diel vertical migration (DVM) simulations.

Species	Forage Depth (m)	Location	Season	Citation

Adélie	6 – 82	Palmer Deep	Summer	Pickett et al 2018[66]
Gentoo	6 – 144	Palmer Deep	Summer	Pickett et al 2018[66]
Chinstrap	25 – 45	South Orkney & King George Island	Summer	Lishman & Crozall 1983[67]; Kokubun et al 2015[68]

Supplemental Movie S1 – Animation of daily average currents within the study region from Dec through March. Currents are depth integrated across the top 150 m and averaged across each model year. Every 25th vector is plotted.
